# Supplementary material for: A Novel Microwave Hot Pressing Machine for Production of Fixed Oils from Different Biopolymeric Structured Tissues
Source: Polymers (Basel). 2023 May 10;15(10):2254. doi: 10.3390/polym15102254 (PMC10224043; doi:10.3390/polym15102254)
Supplement: Supplementary file 1 [file polymers-15-02254-s001.zip › polymers-2234088-supplementary.pdf]

Supplementary Materials for

A novel microwave hot pressing machine for production of fixed oils from different biopolymeric structured-tissues

Sherif S. Hindi [*1]; Uthman M. Dawoud[2]; Iqbal M. *Ismail* [3]; Khalid A. Asiry [1]; Omer H. Ibrahim [1]; Mohammed A. Al-Harthi [1]; Zohair M. Mirdad [1]; Ahmad I. Al-Qubaie [1]; Mohamed H. Shiboob [4]; Najeeb M. Almasoudi [1] and Rakan A. Alanazi [1]

[1] Department of Arid Land Agriculture, Faculty of Environmental Sciences, King Abdullaziz University (KAU), Jeddah 21589, Saudi Arabia

[2] Department of Chemical and Materials Engineering, Faculty of Engineering, KAU, Jeddah 21589, Saudi Arabia

[3] Department of Chemistry, Faculty of Science, and Center of Excellence in Environmental Studies, KAU, P.O Box 80216, Jeddah 21589, Saudi Arabia

[4] Department of Environment, Faculty of Environmental Sciences, KAU, Jeddah 21589, Saudi Arabia

* Correspondence: shindi@kau.edu.sa; +96-656-676-0086.

This PDF file includes:

Section S1. The management plan for testing the microwave-hot pressing machine.

Section S2. The four oily-plants used in the present investigation

Section S3. Synthesis of the fatty acids methyl esters from the four fixed oils.

Section S4. Chemical structure and the mass spectra-chromatogram.

Section S1. The management plan for testing the microwave-hot pressing machine.

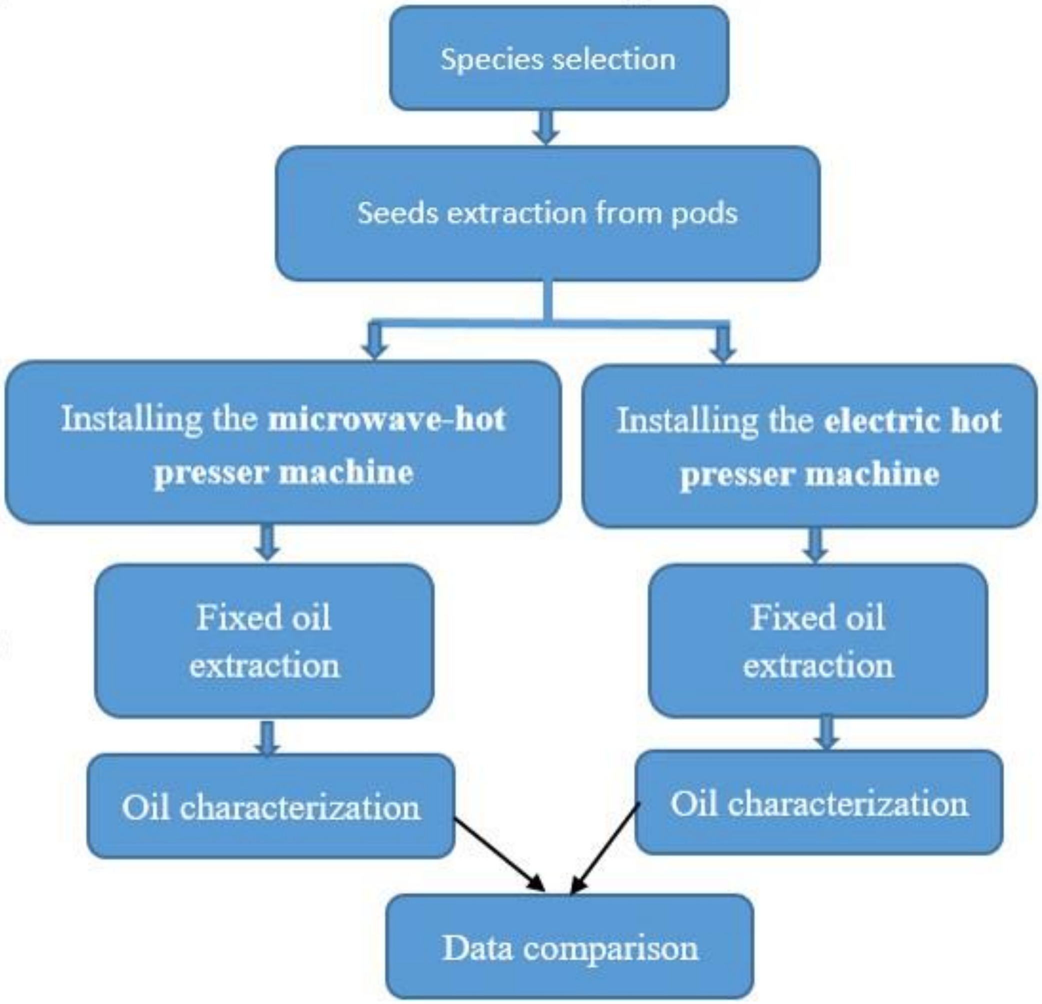

Figure S1. The management plan applied to investigate the efficiency of the microwave-hot pressing machine for fixed oil extraction.

Section S2. Extraction of the four fixed oils.

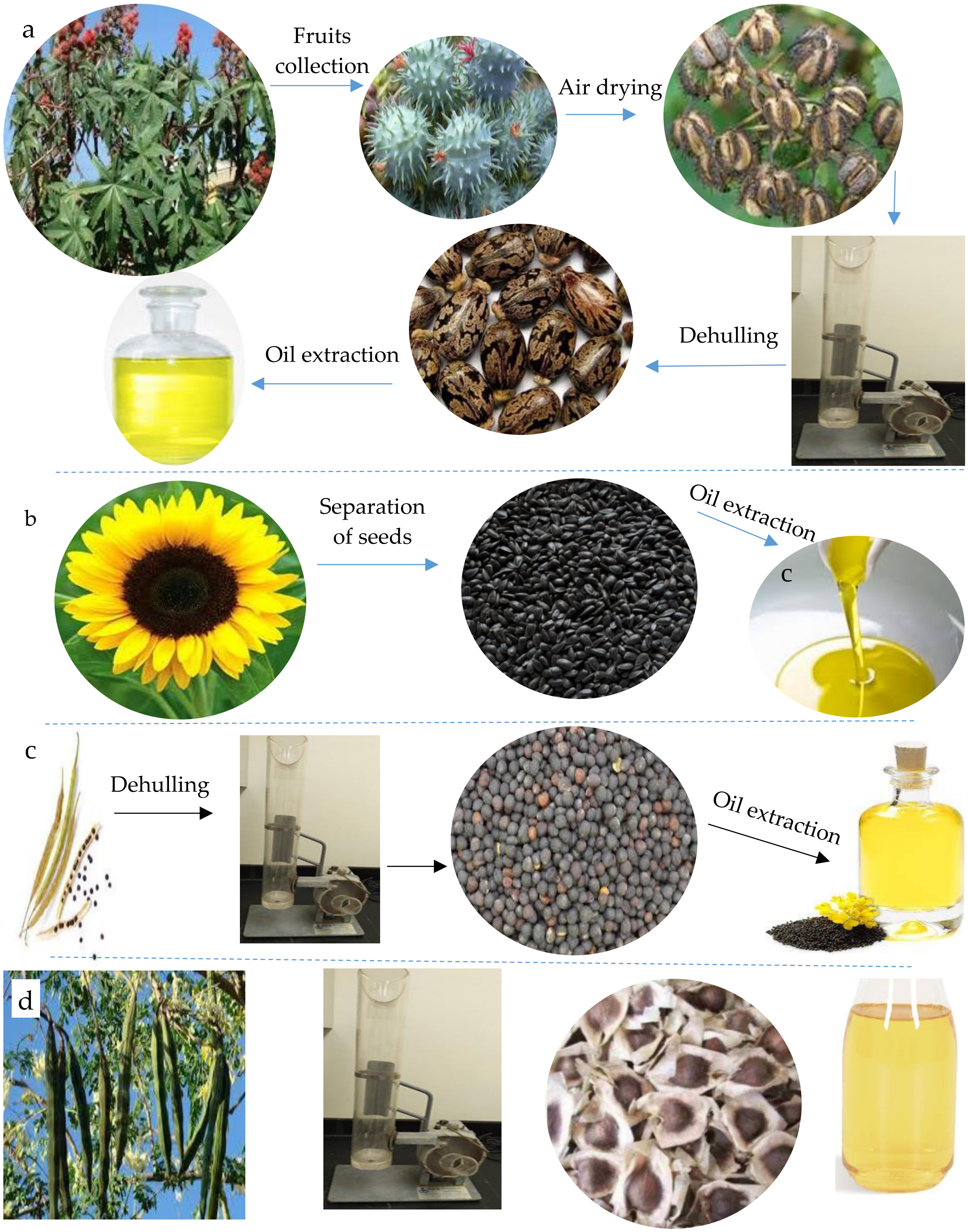

Figure S2. The extraction steps of the four fixed oils from seeds: a) Castor (*Ricinus communis* L.), b) Sunflower (*Helianthus annuus* L.), c) Rapeseed (*Brassica napus* L.), and d) Moringa (*Moringa oleifera* Lam.).

Section S3. Synthesis of the fatty acids methyl esters.

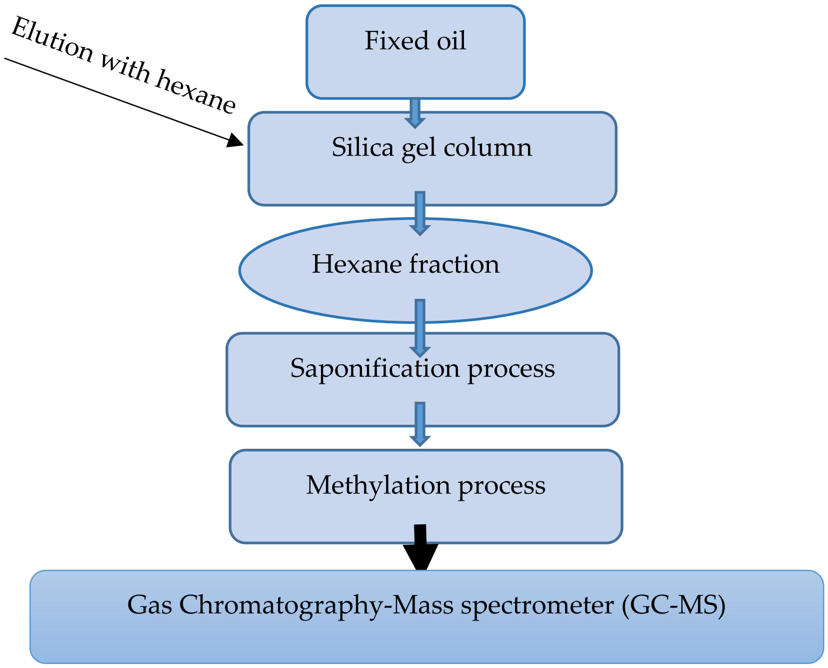

Figure S3. Preparation of fatty acids methyl esters of the four fixed oils to be analyzed by GC-MS.

Section S4. Chemical structure and the mass spectra-chromatogram.

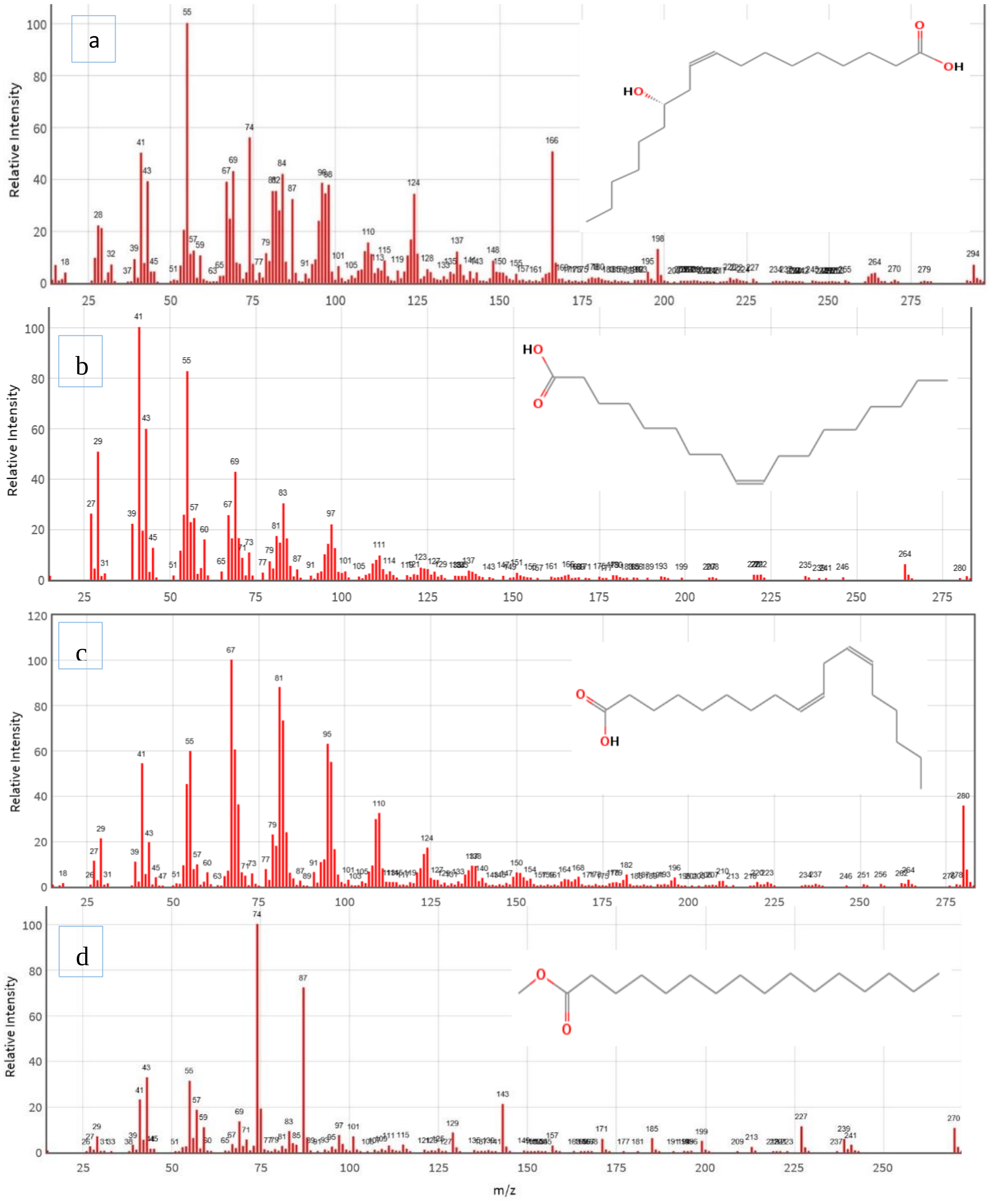

Figure S4. Chemical structure and the mass spectra-chromatogram of: a) Ricinoleic acid methyl ester detected, the main fatty acid of the castor oil, b) Oleic acid, the main fatty acid in the fixed oils of sunflower, rapeseed, and moringa species, c) Linoleic acid detected as the second main fatty acids in the sunflower fixed oil, and d) Palmitic acid detected at the four fixed oils.
